# Considering the protective effect of exendin-4 against oxidative stress in spiral ganglion neurons

**DOI:** 10.22038/IJBMS.2023.69190.15076

**Published:** 2023

**Authors:** Hongxia Wu, Yangxi Ou, Siji Wang, Fenghui Yu, Xiaoxia Fan, Houyong Kang, Tao Chen

**Affiliations:** 1 Department of Otorhinolaryngology, The First Affiliated Hospital of Chongqing Medical Chongqing, China

**Keywords:** Exenatide, Hearing loss, Kanamycin, Mice, Oxidative damage, Spiral ganglio

## Abstract

**Objective(s):**

The protection of spiral ganglion neurons (SGNs) is crucial for hearing loss. Exendin-4 has been shown to have neuroprotective effects in several neurological disorders. Therefore, this study aimed to investigate the effect of the glucagon-like protein-1 receptor (GLP-1R) agonist exendin-4 on kanamycin-induced injury in mouse SGNs *in vitro*.

**Materials and Methods:**

In this study, GLP-1R expression in SGNs was verified by immunofluorescence and immunohistochemical staining. *In vitro*-cultured SGNs and the organ of Corti were exposed to kanamycin with or without exendin-4 treatment. The cell survival rate was measured using the cell counting kit-8 assay, and the damage to auditory nerve fibers (ANF) projecting radially from the SGNs was evaluated using immunofluorescence staining. Reactive oxygen species (ROS) content was determined by flow cytometry, and glutathione peroxidase (GSH-Px) content, superoxide dismutase (SOD) activity, and malondialdehyde (MDA) content were determined by spectrophotometry. Protein expression of nuclear factor erythroid-2-related factor 2/heme oxygenase-1 (Nrf2/HO-1) was detected using western blotting.

**Results:**

GLP-1R was expressed in SGNs. Treatment with 1 mM kanamycin for 24 hr induced SGN damage. Exendin-4 (100 nM) had a protective effect against kanamycin-induced SGN cell injury, improved cell survival rate, reduced nerve fiber injury, increased SOD activity and GSH-Px level, and reduced MDA and ROS contents. The Nrf2/HO-1 pathway was activated.

**Conclusion:**

Exendin-4 alleviates oxidative damage and exerts neuroprotective effects in kanamycin-induced SGN injury through the Nrf2/HO-1 signaling pathway. Exendin-4 has the potential to prevent or treat hearing loss due to SGN damage.

## Introduction

Sensorineural hearing loss (SNHL) is a common type of hearing loss that seriously affects the physical and mental health and quality of life of humans. Although SNHL has a multifactorial etiology, it is mainly associated with damage to spiral ganglion neurons (SGNs) (1, 2). SGNs are the auditory neurons of the inner ear that transmit sound signals from hair cells to the cochlear nucleus in the brain stem (3). SGNs are essential for hearing, and SGN loss can lead to irreversible SNHL. Currently, cochlear implantation is the only effective modality for hearing restoration in patients with severe SNHL, and cochlear implantation requires a certain number of complete and functioning SGNs (4). 

Oxidative stress is a key factor in the development of neurodegenerative diseases (5) and is associated with sensorineural deafness (6). Excess free radical oxygen species (ROS) formation is an important mediator of noise-induced hearing loss (7, 8). Ros-induced lipid peroxidation products lead to decreased blood flow, production of pro-inflammatory cytokines, and cell apoptosis in the cochlea (9). A similar mechanism has been reported for drug-induced hearing loss (10, 11) and aging (12, 13). Glucagon-like peptide-1 (GLP-1) is a polypeptide hormone secreted by intestinal L cells and expressed by the proglucagon gene (14). Some studies have shown that activation of GLP-1/GLP-1 receptor (GLP-1R) signaling prevents memory loss, neuroinflammation, and neurotoxicity (15, 16). The neuroprotective effects of GLP-1R agonists and their exact mechanisms have been studied in Alzheimer’s disease, Parkinson’s disease, vascular brain injury, epilepsy, and other neuropathological diseases (14, 17, 18). However, whether GLP-1R is expressed in the inner ear nerve cells and whether GLP-1R agonists affect the inner ear nerve cells have not been elucidated.

However, there are no effective drugs for the treatment of SGN injuries. Therefore, the development of effective drugs to prevent SGN damage and promote SGN repair can provide new treatment and prevention strategies for SNHL. Kanamycin, is one of the aminoglycoside antibiotics, although aminoglycoside antibiotics have beneficial effects in antibacterial therapy, they can cause irreversible sensorineural hearing loss (19). Aminoglycoside-induced hearing loss involves oxidative stress and inflammatory responses (20). Kanamycin-induced hearing loss was associated with oxidative stress, inflammation, and increased expression of genes associated with apoptosis (21). Thus, this study aimed to investigate the effect of the GLP-1R agonist exendin-4 on kanamycin-induced injury in mouse SGNs *in vitro*. 

## Materials and Methods

***Materials***

Dulbecco’s Modified Eagle medium (DMEM) with Ham’s F-12 and 10% fetal bovine serum were purchased from GIBCO (California, USA). Kanamycin monosulfate was purchased from Coolabeling (Beijing, China). Exendin-4 (exenatide) was purchased from MedChemExpress (New Jersey, USA), and the cell counting kit-8 (CCK-8) was obtained from Dojindo Laboratories (Kumamoto, Japan). The reactive oxygen species (ROS) assay kit was purchased from Beyotime Biotechnology (Shanghai, China). The primary antibodies used in this study were anti-GLP-1R (ab218532, Abcam, Cambridge, MA, USA) ,anti-heme oxygenase-1 (HO-1,Abcam,ab189491), anti-nuclear  factor erythroid-2-related factor 2 (Nrf2; Abcam, ab62352), and anti-β-actin ( Proteintech,20536-1-AP). The primary antibody anti-neurofilament-H (anti-NF-H) was purchased from Cell Signaling Technology (#2836S, Danvers, MA, USA). Horseradish peroxidase (HRP)-labeled secondary antibody, goat anti-mouse immunoglobulin (IgG)-biotin secondary antibody, and goat anti-rabbit IgG-biotin secondary antibody were purchased from ZENBIO (Beijing 4A Biotech Co., Ltd., Beijing, China). As secondary antibodies, Alexa Fluor 594-labeled goat anti-rabbit IgG-biotin and Alexa Fluor488-labeled goat anti-mouse IgG-biotin were obtained from ZSBIO (Beijing, China). Glutathione peroxidase (GSH-Px), superoxide dismutase (SOD), and malonaldehyde (MDA) assay kits were purchased from the Nanjing Jiancheng Bioengineering Institute (Nanjing, China). A bicinchoninic acid protein assay kit was purchased from Beyotime Biotechnology (Shanghai, China).

***Animals***

C57BL/6J mice were purchased from the Laboratory Animal Center of Chongqing Medical University (Chongqing, China). None of the animals had a history of ototoxic damage or noise exposure, and all were housed under standard laboratory conditions (temperature was 22±1 °C, the daily light time was 8:00 am-8:00 pm, and the humidity was 55%). All animal care and experimental procedures were approved by the appropriate ethics committee (Approval No.: 2022-K211). All applicable international, national, and/or institutional guidelines for the care and use of animals were followed.

***Primary culture of spiral ganglion neurons***

C57BL/6J mice were sacrificed 3–5 days after birth. Briefly, mice were anesthetized using ether inhalation and sterilized with 75% ethanol. Then, an incision was made at the base of the foramen magnum, along the sagittal cranial suture, and brain tissue was removed. The following steps were performed under a microscope and in pre-cooled phosphate buffer solution (PBS). The bulla of the temporal bone was opened, and the capsule of the inner ear, stria vascularis, and organ of Corti were removed. The Rosenthal canal was isolated and digested with 0.25% trypsin for 30 min at 37 °C. After centrifugation at 1000 rpm for 5 min, the cell suspensions were placed on 35-mm cell culture plates coated with poly-L-lysine. Cells were maintained in DMEM/F12 medium supplemented with 10% fetal bovine serum, 5% neurotrophic factor B27, and 2% penicillin and then placed in an incubator at 37 °C under 5% CO_2_ and 95% air. All animal experiments were reported in detail in previous studies (22).

***Immunofluorescence***

SGNs were cultured as mentioned above. Briefly, the cells were grown in 24-well plates, washed twice with phosphate buffer (PBS), and fixed with 4% paraformaldehyde at room temperature (20–30 °C) for 15 min. Then, the cells were treated with 0.25% Triton X-100 for 15 min and blocked with 10% normal goat serum for 30 min. Thereafter, the cells were incubated overnight with anti-GLP-1R (1:200 dilution) and anti-neurofilament H (1:200 dilution) primary antibodies at 4 °C. The control group was administered PBS instead of a primary antibody. After washing three times with PBS, the cells were stained with the secondary antibodies Alexa Fluor-labeled goat anti-rabbit IgG 594 (1:500 dilution) and Alexa Fluor488-labeled goat anti-mouse IgG (1:500 dilution). The mixture was allowed to stand at room temperature for 1 hr. After the tablets were sealed with an anti-fluorescence quenching solution containing DAPI, they were observed under a ZEISS fluorescence microscope.

***Mouse cochlea section and immunohistochemical analysis***

Adult C57BL/6J mice were anesthetized with pentobarbital and sacrificed. The temporal bone tissue was removed, decalcified with EDTA solution for 7 days, embedded in paraffin, and sectioned. The sections were then placed in an oven at 60 ° for 3 hr; soaked in xylene solution twice for 15 min each time; and then soaked in 95% ethanol, 85% ethanol, 75% ethanol, and 50% ethanol successively for 5 min each time. Then, 4% hydrogen peroxide was added, and the sections were soaked for 10 min. The sections were then incubated with 10% normal goat serum for 30 min, with added anti-neurofilament-H (1:100 dilution) primary antibody, and incubated at 4 °C overnight. After three times washing with PBS, the HRP-labeled goat anti-mouse secondary antibody (1:200 dilution) was added, and the sections were incubated at room temperature for 1 hr. Consequently, the sections were re-dyed with hematoxylin for 5 min and then differentiated with hydrochloric acid alcohol for 2 sec and ammonia anti-blue for 2 min. Thereafter, they were successively soaked in 75% alcohol, 85% alcohol, and 95% alcohol for 5 min each time and then soaked twice in xylene solution for 15 min each time. The tablets were sealed with a neutral resin-based sealing solution. All tablets were observed and imaged under a ZEISS microscope.

***In vitro culture and immunofluorescence staining of the organ of corti***

Round Cover Slips coated with poly-L-lysine were placed in culture dishes. DMEM/F12 culture medium containing 10% fetal bovine serum and 2% penicillin was then added, and the plates were placed in an incubator at 37 °C under 5% CO_2_ and 95% air for 30 min. After dissection following the above method, the inner ear tissue was removed, the organ of Corti was isolated, and the position was adjusted under the microscope so that it was fully unfolded and attached to the cell crawling plate. Then, the tissue was placed in an incubator at 37 °C under 5% CO_2_ and 95% air for culture. 

For immunofluorescence staining, after 3 days of culture, the organ of Corti was treated with kanamycin (1 mM) and with or without exendin-4 (100 nM) for 24 hr. The cells were fixed with 4% paraformaldehyde for 2 hr and gently washed three times with PBS. They were then incubated with PBS containing 10% Triton X100 and 10% goat serum for 2 hr. Anti-neurofilament H (1:200 dilution) primary antibody containing 0.2% Triton X100 and 10% goat serum was added, and the cells were incubated at 4 ℃ for 24 hr. After washing three times with PBS, the secondary antibody Alexa Fluor488-labeled goat anti-mouse IgG fluorescent (1:100) containing 0.2% Triton X100 and 10% goat serum was added, and the cells were incubated at room temperature ( 20–30 °C) for 1 hr for staining. The cells were then observed under a ZEISS fluorescence microscope.

***Drug treatment***

Exendin-4 is soluble in dimethyl sulfoxide at concentrations that do not exceed 0.1% of the total cell volume (according to the scheme provided by the manufacturer). On day 3 of cell culture, SGNs were pretreated with exendin-4 (dosing was given for each result), and kanamycin was added to the culture solution (dosing was given for each result).

***Cell viability assay***

SGNs were cultured in 96-well plates at a density of 5×10^4^ cells/well at 37 °C until they achieved 80% density. Cell survival was measured using the CCK-8 kit according to the manufacturer’s protocol. The optical density (OD) was measured with an enzyme-linked immunoassay at 450 nm. Relative cell viability was calculated using the following formula: relative cell viability=[OD (experimental group) – OD (blank group))/(OD (control group) – OD (blank group)]×100%.

***Flow cytometry of reactive oxygen species content***

SGNs were co-incubated with kanamycin (1 mM) for 24 hr and treated with or without (control) exendin-4 (100nM). ROS content post-treatment was measured using the ROS assay kit according to the manufacturer’s protocol. The mean fluorescence intensity was detected using flow cytometry after the cells were digested with 0.25% trypsin and washed with PBS.

***Spectrophotometric detection of GSH-Px content, SOD activity, and MDA level***

SGNs were co-incubated with kanamycin (1 mM) for 24 hr and treated with or without (control) exendin-4 (100nM). The cells were then washed twice with cold PBS, collected, and lysed using ultrasound for subsequent testing. The levels of GSH-Px, SOD, and MDA in each group were detected using commercial kits, according to the manufacturer’s instructions. All experiments were repeated thrice.

***Western blot assay***

After treating the SGNs with or without exendin-4 (100 nM) and kanamycin (1 mM) for 24 hr, cell proteins were collected, and the protein content of the samples was determined using the BCA protein analysis kit. Protein was separated using 10% SDS-PAGE. The separated proteins were electrically transferred to a nitrocellulose membrane and sealed at room temperature with a rapid sealing solution for 30 min. Next, target primary antibodies were added, and the samples were incubated overnight at 4 °C. After washing three times with TBST, the samples were added a secondary antibody and incubated at room temperature for 1 hr. Western blotting was performed using an enhanced ECL kit (ZENBIO, China) with β-actin as the internal control. The band intensities were estimated using the EvolutionCapt software package (VILBER BIO IMAGING, Paris, France).

***Statistical analysis***

Data were analyzed using one-way analysis of variance (ANOVA) with the SPSS 13.0 software package (IBM, New York, USA). The histograms were drawn using GraphPad Prism 8 (GraphPad Software, California, USA). A *P*-value of <0.05 was considered significant.

## Results

***GLP-1R is expressed in SGNs***

**Figure 1:**
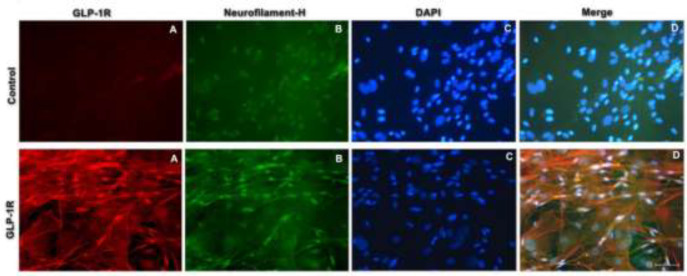
Fluorescence of the primary culture and corresponding photograph showed that GLP-1R was expressed in SGNs

**Figure 2:**
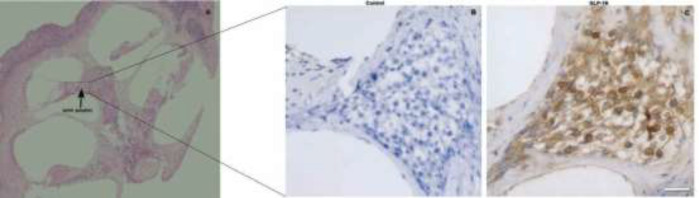
Immunohistochemical staining of paraffin sections of mouse cochlea demonstrating GLP-1R expression in SGNs

Exendin-4 is a specific GLP-1R receptor agonist (23), however, data on GLP-1R expression in SGNs are rare. As shown in Figure 1a, DAPI-tagged nuclei appeared blue under UV light and SGNs appeared to fluoresce green under blue light after being tagged with neurofilament-H. GLP-1R-expressing cells showed fluoresce red under green light. Under the same field of view, GLP-1R was expressed in SGNs after excitation with green light, blue light, and ultraviolet light (Figure 1). As shown in Figure 1b, SGNs in osseous spiral lamina labeled by anti-GLP-1R appear brownish yellow (Figure 2).

***Exendin-4 exerts a protective effect on kanamycin-induced SGN injury***

**Figure 3:**
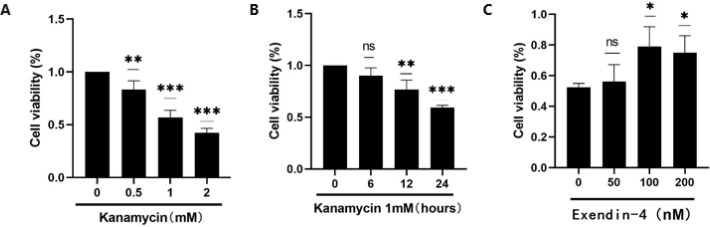
Exendin-4 has antagonistic effect on kanamycin-induced SGNs damage

SGNs were exposed to different concentrations of kanamycin (24) for different time periods. SGNs treated with 1 mM concentration for 24 hr showed lower cell viability than the control group. Thus, this concentration and treatment time were used for the follow-up experiments. After pretreatment with different concentrations of exendin-4 (50, 100, and 200 nM) for 3 hr (25-27), SGNs were exposed to 1 mM kanamycin and co-treated with exendin-4 for 24 hr. Comparison of cell survival showed that exendin-4 counteracted the toxicity of kanamycin to SGNs, and the neuroprotective effect was strongest at exendin-4 concentration of 100 nM. However, exendin-4 did not show a protective effect beyond a dose of 200 nM, and cell viability decreased (Figure 3).

**Figure 4:**
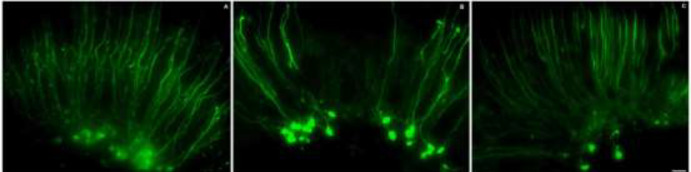
Kanamycin-induced ANF damage was reduced by exendin-4

The organ of Corti was incubated with kanamycin (1 mM) for 24 hr and then treated with or without exendin-4 (100 nM). Neurofilament-H labeling showed that a large number of ANF were lost in the kanamycin (1 mM) treatment group or were significantly reduced after exendin-4 (100 nM) treatment (Figure 4). 

***Exendin-4 mitigated kanamycin-induced oxidative damage on SGNs***

**Figure 5:**
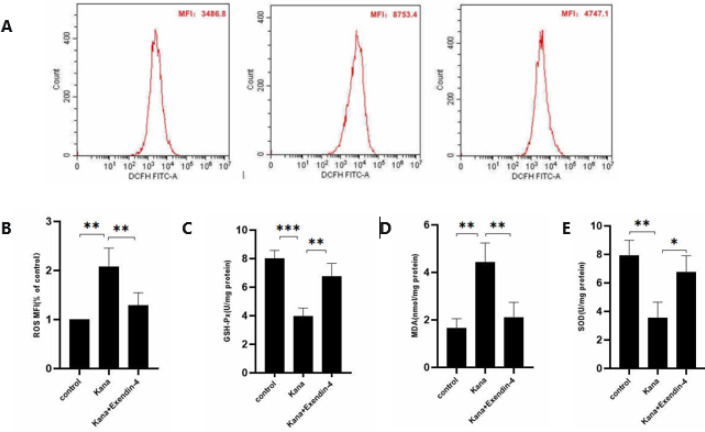
Exendin-4 (100 nM) treatment could effectively antagonize the oxidative damage of SGNs induced by kanamycin (1 mM)

Previous studies have shown that kanamycin-induced damage to SGNs may be related to oxidative stress (28), and GLP-1R agonists can reduce the damage caused by oxidative stress (29-31). Therefore, we examined whether the GLP-1R agonist exendin-4 antagonized kanamycin-induced SGN damage by alleviating the oxidative stress response. The ROS content in the kanamycin (1 mM) group was significantly increased more than that in the control group, and the addition of exendin-4 (100 nM) reduced the ROS content (Figure 5A-5B). Furthermore, after kanamycin (1 mM) treatment, the GSH-Px content decreased compared with the control group, whereas the addition of exendin-4 (100 nM) reversed the kanamycin-induced decrease in GSH-Px content (Figure 5C). 

Compared with the control group, the kanamycin treatment group showed significantly higher MDA levels. Overall, exendin-4 (100 nM) reduced MDA content (Figure 5D). The SOD activity of the kanamycin treatment group was significantly decreased compared with the control group. The SOD activity of SGNs treated with exendin-4 (100 nM) was significantly higher compared with the kanamycin treatment group (Figure 5E). All differences were statistically significant. The above results indicated that exendin-4 could effectively antagonize the kanamycin-induced decrease in GSH-Px content and SOD activity and reduce the kanamycin-induced increase in ROS and MDA. 

Exendin-4 may alleviate kanamycin-induced oxidative damage through the Nrf2/HO-1 pathway.

**Figure 6:**
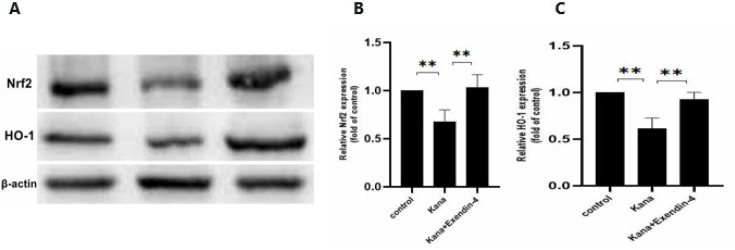
Effect of exendin-4 on the protein expression of Nrf2 and HO-1

Nrf2 is a transcription factor that protects against oxidative stress-related diseases (32,33). Under oxidative damage, Nrf2 undergoes nuclear translocation and activation to activate the transcription of downstream anti-oxidant genes, including superoxide dismutase and HO-1, thereby reducing oxidative damage (34, 35). In western blot analysis, SGNs exposed to 1 mM kanamycin for 24 hr showed decreased protein expression of Nrf2 and HO-1. In contrast, SGNs treated with exendin-4 (100 nM) showed significantly increased expression levels of Nrf2 and HO-1(Figure 6). These results suggest that exendin-4 may alleviate kanamycin-induced oxidative damage by activating the Nrf2/HO-1 pathway.

(A) GLP-1R showing red fluorescence. (B) Neurofilament-H tagged SGNs. (C) DAPI staining of the nuclei. (D) A-C merged with the same vision. Scale bar: 10 µm

(A) Cochlear biopsy (HE staining; →:spiral ganglion; : osseous spiral lamina). (B) Control group treated with PBS instead of primary antibody. (C) SGNs in osseous spiral lamina were labeled by anti-GLP-1R and appear brownish yellow.

Scale bar: 50 µm

The cell survival rate is determined using the CCK-8 assay. (A) Kanamycin (0, 0.5, 1, and 2 mM) treatment for 24 hr induces SGN injury and decreases cell survival rate. (B) Kanamycin treatment (1 mM; 0, 6, 12, and 24 hr) induces SGN injury and decreases cell survival. (C) The survival rate of SGN cells is increased after co-treatment with exendin-4 (0, 50, 100, and 200 nM) and kanamycin (1 mM) for 24 hr

*P<0.05, **P<0.01, and ***P<0.001, relative to the control

Immunofluorescence image of the organ of Corti treated with 1 mM kanamycin for 24 hr with or without exendin-4 (100 nM). (A) Untreated control group. (B) The kanamycin (1 mM) treatment group showed loss of a large number of ANF. (C) Loss of ANF is significantly reduced after the addition of exendin-4 (100 nM).

Scale bar: 10 µm

In comparison with the control group, the kanamycin (1 mM) group shows higher ROS production, lower GSH-Px content, lower SOD activity, and higher MDA content. Exendin-4 (100 nM) reverses these changes. (A, B) ROS content. MFI: Mean fluorescence intensity (C) GSH-Px content. (D) MDA content. (E) SOD activity.

Kana: kanamycin; *P<0.05, **P<0.0.1, and ***P<0.0.01

In comparison with the untreated control group, the kanamycin group shows significantly lower expressions of Nrf2 and HO-1. After the exendin-4 treatment, the expression levels of Nrf2 and HO-1 are significantly higher than in the kanamycin group.

*P<0.0.5, **P<0.0.1, and ***P<0.0.01

## Discussion

In this study, the expression of GLP-1R in SGNs was verified. GLP-1R was first discovered in rat islet cells in 1987(36). Soon after, it was localized to various tissues, including the pancreas, lungs, brain, stomach, heart, kidney, and other tissues in humans and mice (37). Our study demonstrates the expression of GLP-1R in SGNs.GLP-1 acts in combination with GLP-1R. GLP-1/GLP-1R reduces ischemia-induced damage by inhibiting apoptosis and oxidative stress, providing cardiac protection and reducing the size of heart infarcts in rats (38). GLP-1/GLP-1R also plays a neuroprotective role in cerebral ischemia (39), craniocerebral trauma (40), Alzheimer’s disease (41), Parkinson’s disease (42), and many other neurodegenerative diseases (43). Due to the short half-life of natural GLP-1 (44), its clinical application is limited, so researchers have developed a variety of GLP-1 analogs, Exendin-4(exenatide) being one of them. Exendin-4 is a polypeptide comprising 39 amino acids that is a long-acting glucagon-like peptide-1 receptor agonist. Many studies have shown that exenatide can play a neuroprotective role in a variety of diseases (45-48). Therefore, we supposed that exendin-4 may also have a protective effect on the nerve cells of the inner ear.

In subsequent experiments, SGNs treated with kanamycin (1 mM) showed significantly reduced cell survival after 24 hr, as confirmed by the CCK-8 assay. It is generally believed that the loss of SGNs is usually secondary to the loss of hair cells (HCs), however, some studies have shown that SGNs can also be damaged or lost without HCs damage ( 49). Previous studies have reported that kanamycin causes a retraction of auditory nerve terminals from hair cells, suggesting that kanamycin may have a direct neurotoxic effect on SGNs (50). This is consistent with our results. Meanwhile, the results of CCK-8 showed that exendin-4 (100 nM) significantly reduced kanamycin-induced SGNs damage. However, when the concentration of exendin-4 reached 200 nM, it did not show a stronger protective effect and cell survival was decreased. This result may be due to the cytotoxicity caused by the high concentration of exendin-4.

*In vitro* treatment of the organ of Corti with kanamycin (1 mM) and exendin-4 (100 nM) showed that kanamycin (1 mM) treatment resulted in the loss of a large number of ANF. This is consistent with previous research results ( 51). In contrast, exendin-4 (100 nM) could antagonize this toxic effect. Collectively, these results suggested that exendin-4 at appropriate concentrations can protect SGNs from kanamycin damage.

And then the results of the experiment demonstrated that exendin-4 can increase GSH-Px content and SOD activity and decrease MDA and ROS contents, suggesting that exendin-4 can enhance the anti-oxidant activity of bioactive molecules in SGNs and alleviate kanamycin-mediated oxidative stress. Oxidative stress is associated with sensorineural hearing loss caused by factors such as loud noise (52), ototoxic drugs (53), radioactive radiation (54), ischemia, and aging (55) and is a key factor in neurodegeneration (5). Previous studies have shown that kanamycin-induced damage to SGNs is related to the oxidative stress response (28), while studies in other tissue cells have shown that GLP-1R agonists can alleviate oxidative stress-induced damage (29-31). Therefore, we examined whether the GLP-1R agonist exendin-4 antagonized kanamycin-induced SGN damage by alleviating the oxidative stress response. Glutathione peroxidase (GSH-Px) is an important peroxidase-decomposing enzyme that is widely present in organisms and is an important component of anti-oxidants. SOD is an oxygen-free radical scavenger that can transform superoxide anion free radicals upstream of the reactive oxygen metabolism cascade to protect cells from damage. MDA is an important product of membrane lipid peroxidation. ROS are highly reactive chemicals that contain free oxygen radicals. MDA and ROS levels are significantly increased in oxidative stress reactions, which are important markers of oxidative stress reactions (27-29). And our findings are consistent with previous reports that exendin-4 can inhibit oxidative stress responses in other cells (56-58). 

The results of Western blot showed that kanamycin inhibited the expression of Nrf2/HO-1, while exendin-4 significantly reversed this result and activated protein expression of Nrf2/HO-1. Regulation of the Nrf2/HO-1 pathway is considered a key adaptive system for maintaining cell REDOX homeostasis and enhancing cell resistance to oxidative stress (32-35). Studies have shown that GLP-1R agonists can activate the Nrf2/HO-1 pathway and reduce cellular oxidative damage (59, 60). We suggested that exendin-4 may protect SGNs from kanamycin-induced oxidative damage by activating the Nrf2/HO-1 pathway.

In summary, our study showed that the GLP-1R receptor is expressed in SGNs of the inner ear, and the GLP-1R agonist exendin-4 can alleviate the oxidative stress response and antagonize kanamycin-induced SGN damage by activating the Nrf2/HO-1 pathway. This effect requires further investigation in animal experiments.

## Conclusion

Exendin-4 plays a role in alleviating oxidative damage and exerts neuroprotective effects in kanamycin-induced SGN damage through the Nrf2/HO-1 signaling pathway. Exendin-4 and other GLP-1R agonists are promising acoustic neuroprotective agents, and exendin-4 has the potential to prevent or treat hearing loss due to SGN damage.

## Authors’ Contributions

HX W and T C designed the experiments; HX W, YX O, SJ W, FH Y, and XX F performed experiments and collected data; HX W and T C discussed the results and strategy; T C and HY K supervised, directed, and managed the study; T C and HY K approved the final version to be published.

## Conflicts of Interest

The authors declare no conflicts of interest.
